# Development of a Novel, Low-Cost, Disposable Wooden Pencil Graphite Electrode for Use in the Determination of Antioxidants and Other Biological Compounds

**DOI:** 10.3390/s150818887

**Published:** 2015-07-31

**Authors:** James Kariuki, Emily Ervin, Carly Olafson

**Affiliations:** Department of Science, Augustana Campus, University of Alberta, 4901-46th Ave, Camrose, AB T4V 2R3, Canada; E-Mails: eervin@ualberta.ca (E.E.); caolafso@ualberta.ca (C.O.)

**Keywords:** antioxidant, biosensor, glassy carbon electrode, pencil graphite electrode, square-wave voltammetry

## Abstract

The development of portable sensors that can be used outside the lab is an active area of research in the electroanalytical field. A major focus of such research is the development of low-cost electrodes for use in these sensors. Current electrodes, such as glassy-carbon electrodes (GCEs), are costly and require time-consuming preparation. Alternatives have been proposed, including mechanical pencil-lead electrodes (MPEs). However, MPEs themselves possess numerous drawbacks, particularly structural fragility. In this paper, we present a novel pencil-graphite electrode (PGE) fabricated from a regular HB#2 pencil. This PGE is a simple, disposable, extremely low-cost alternative to GCEs ($0.30 per PGE, *vs.* $190 + per GCE), and possesses the structural stability that MPEs lack. PGEs were characterized by square-wave voltammetry of ferricyanide, gallic acid, uric acid, dopamine, and several foodstuffs. In all cases, PGEs demonstrated sensitivities comparable or superior to those of the GCE and MPE (LOD = 5.62 × 10^−4^ M PGE, 4.80 × 10^−4^ M GCE, 2.93 × 10^−4^ M MPE). Signal areas and peak heights were typically four to ten times larger for the PGE relative to the GCE.

## 1. Introduction

The chemical reactivity of the working electrode (where the reaction of interest occurs) has significant impact on the function of electrochemical sensors. Currently, the glassy carbon electrode (GCE) is the most commonly employed working electrode material due to the ability of carbon to react with diverse classes of analyte [1,2]. In the field of antioxidant research, GCEs have been used to determine the antioxidant capacity of dried vegetable extracts [3], wines [4], fruit and vegetable samples [5,6], blood plasma [7], tea infusions [8], mushroom extracts [9], and standard phenolic and flavonoid solutions [10,11,12]. However, despite their widespread usage, GCEs are prone to electrode fouling, are relatively costly (approximately $190 to $1200 each, depending on the exact specifications and supplier) [13,14], and require labour-intensive and time-consuming cleaning procedures [2,15,16].

Numerous modifications and novel electrode materials have been proposed to overcome the limitations of traditional glassy carbon-based sensors, including metallization, derivatization and doping [17]. In particular, however, interest has turned to the development of pencil-graphite electrodes (PGEs), due to their disposability, simplicity and low cost [16]. PGEs derived from mechanical pencil leads (MPEs) have successfully been used to analyze vitamin B12 [18,19], the anti-tapeworm drug niclosamide [20], the copper-chelating properties of flavonoids [2], the hybridization of DNA [21], the anti-cancer drug paclitaxel [22], uric acid and dopamine in urine and blood plasma [23,24,25], glucose [26], an organophosphorus pesticide [27], hydrazine [28], and capsaicin [29]. However, such MPEs themselves possess numerous disadvantages, including structural fragility and the necessity of a specialized holding device. Furthermore, because each lead can only be used for a few scans, MPEs introduce error as the same electrode cannot be used for an entire experimental series.

In this paper, we present a fully developed pencil electrode derived from a regular HB #2 pencil. Building upon previous preliminary characterizations of graphite and pencil electrodes conducted by one of the authors [16], this wooden PGE has been specifically designed to be disposable, simple to fabricate, rapid to use, structurally stable, sensitive, and extremely cost effective; as such this PGE overcomes many of the disadvantages of both GCE and MPE sensors.

In addition to describing the development of the wooden pencil graphite sensor, this paper will demonstrate several biological applications of the electrode, including: gallic acid determination; determination of total antioxidant capacity in samples of fruits, vegetables, tea, and coffee; determination of uric acid; and determination of dopamine. As such, we hope to establish the developed sensor as a novel, extremely cost-effective, structurally stable and highly sensitive alternative to traditional GCEs and MPEs.

## 2. Experimental Section

### 2.1. Reagents

Ferricyanide and gallic acid were obtained from Fisher Scientific (Ottawa, ON, Canada). Anhydrous ethanol was obtained from Commercial Alcohols (Brampton, ON, Canada), uric acid and dopamine from Sigma-Aldrich (St. Louis, MO, USA), potassium chloride from J. T. Baker (Center Valley, PA, USA) and Scotchgard^®^ from Canadian Tire (Camrose, AB, Canada). Dixon^®^ Economiser HB#2 pencils were purchased from Walmart (Camrose, AB, Canada) and 0.7 mm HB#2 Paper Mate^®^ mechanical pencil leads from Staples Inc. (Camrose, AB, Canada).

### 2.2. Preparation of PGEs

Dixon^®^ Economiser HB #2 pencils were used as the base material for the developed PGEs. HB#2 pencils were chosen as previous research conducted by one of the authors revealed HB#2 graphite to possess electron transfer rates more similar to GC than any other graphite hardness when tested with the redox benchmark reagents of ferricyanide and ruthenium hexammine (other types analyzed included 4B, 2B, 2H, and 4H) [16]. More details of the graphite surface characterization are given in a previous report [16]. The eraser was detached, and the wood casing removed using a knife until the pencil could be easily passed through a 5 mm in diameter hole. The pencils were then smoothed with 150-grit sandpaper, before being treated with Scotchgard (SG), a hydrophobic coating. SG was applied according to manufacturer’s directions. Briefly, PGEs were arrayed vertically in a Styrofoam holder and treated on all surfaces with the SG aerosol, using two light coats. After curing the first coat for 6 h at room temperature (25 ± 1 °C), a second coat was applied, after which the PGEs were left to cure overnight. Finally, the wood from the top 1 cm of the pencil was carefully removed using a small razor blade, until the pencil lead was fully exposed. The lead was then rinsed successively with acetone and distilled water to remove any traces of adhesive; this exposed surface served as the point of attachment of the PGE to the potentiostat. 

### 2.3. Instrumentation

Square-wave voltammetry (SWV) was performed using a model AFCBP1 bipotentiostat (Pine Instrument Company, Durham, NC, USA) and AfterMath^TM^ software. Electrochemical testing was carried out in a three-electrode cell using a Pt counter and a Ag/AgCl reference electrode. Prior to each run, the Pt counter electrode was polished on a wetted Microcloth^®^ pad (Lake Bluff, IL, USA) and rinsed with distilled water, while the working electrodes were prepared as described in Section 2.3.1, Section 2.3.2 and Section 2.3.3.

#### 2.3.1. PGE Polishing

Prior to the analysis of each solution, a 2–3 mm end section of the PGE was removed using a razor blade, and the exposed PGE tip polished on 150-grit sandpaper until a substantial amount of graphite was removed, before being polished on a wetted Buehler Microcloth^®^ polishing pad. The PGE was then rinsed with anhydrous ethanol, followed by distilled water, before being immediately tested. Before each run in the same solution, the PGE was re-polished on 150-grit sandpaper, then polished on a wetted Buehler Microcloth^®^ polishing pad, before being successively rinsed with anhydrous ethanol and distilled water.

#### 2.3.2. MPE Polishing

Prior to analysis, the 0.7 mm Paper Mate^®^ lead was rinsed with anhydrous ethanol followed by distilled water. Exactly 9 mm of the lead was then submerged in the test solution and analyzed by SWV. Following each run, the used portion of the lead was removed using a razor blade, and a new section of the lead exposed for the subsequent run.

#### 2.3.3 GCE Polishing

Prior to each run, the GCE was consecutively polished on a Buehler Microcloth^®^ pad with aqueous slurries of 1.0, 0.3 and 0.05 micron Buehler Micropolish II^®^ alumina powder. Following each round of polishing, the GCE was rinsed and sonicated in distilled water for 5 min. After the final polish, the GCE was sonicated in distilled water for 5 min, followed by sonication in anhydrous ethanol for 5 min, after which the electrode was rinsed with distilled water and immediately analyzed.

### 2.4. Ferricyanide Characterization

The PGE, MPE, and commercial GCE were each analyzed in 1 mM ferricyanide/0.1 M KCl (with distilled water as the solvent) by cyclic voltammetry (CV) (n = 4). The oxidation and reduction peaks of each curve were recorded, and analyzed to determine the ∆Ep of each electrode type. CV parameters: initial potential = 0.7 V; vertex potential = −0.1 V; final potential = 0.7 V; scan rate = 0.05 V/s.

### 2.5. Gallic Acid (GA) Calibration Curve

A SWV calibration curve of peak area as a function of GA concentration was prepared for the PGE, MPE, and GCE (concentration of GA = 8 × 10^−3^ M to 4 × 10^−5^ M, n = 4). GA standards were prepared using 0.1 M, pH 2.0 phosphate buffer with 0.1 M KCl as the supporting electrolyte. For each curve, the potential at the maximum peak current as well as the area from 0.2 to 0.7 V was recorded. The signal given by the area under the peak was chosen as the empirical unit of measurement. The area of a voltammogram is indicative of the antioxidant capacity of the sample, a thermodynamic measurement of antioxidant efficiency which is indicative of the antioxidant concentration; a greater area equates to a greater antioxidant capacity, and hence a greater concentration [7,8,30,31]. SWV parameters: initial potential = 0.2 V; final potential = 0.7 V; amplitude = 0.1 V; period = 100 ms; increment = 0.005 V; sampling width = 16.7 ms. 

### 2.6. Fruit and Vegetable Analysis

Prepared PGEs and GCEs were tested in extracts of carrot, tomato, peach and apple. Extracts were prepared by blending a 25 g sample with 50 mL 0.1 M, pH 2.0 phosphate buffer/0.1 M KCl, and suction filtering the resultant mixture through No. 4 Whatman filter paper. The filtrates were then tested by SWV (n = 3), using the same parameters used for the GA calibration curve. 

### 2.7. Hot Beverage Analysis

Samples of coffee (Gevalia^®^ Kaffe Dark House Blend) and tea (Tetley^®^ Pure Green Tea) were prepared using a Tassimo^®^ T20 automatic brewer. Two T-Discs^®^ of each were brewed in accordance with the barcode specifications. The prepared beverages were then cooled to room temperature. 10 mL of each beverage was then transferred to a 25 mL beaker with 0.07 g KCl, to give a 0.1 M KCl supporting electrolyte. In turn, each of the prepared solutions was then analyzed four times by SWV. Parameters: Ei = 0 V; Ef = 0.9 V; amplitude = 0.1 V; period= 100 ms; increment = 0.005 V; sampling width = 16.7 ms (= 60 Hz); range = 250 µA (coffee), 150 µA (tea).

### 2.8. Uric Acid and Dopamine

For proof of principle, and to show the utility of the developed PGE in the determination of biological analytes, 0.00001 M samples of uric acid (UA) and dopamine (DA) were prepared and analyzed by SWV (n = 3) using both a GCE and a PGE, and the results were compared. Parameters: Ei = 0.2 V; Ef = 0.7 V; amplitude = 0.1 V; period= 100 ms; increment = 0.005 V; sampling width = 16.7 ms (= 60 Hz); range = 100 µA. The voltammograms for both UA and DA were well defined, even at the low concentrations used, and showed distinct oxidation peaks that enable an accurate measurement of peak areas. This is an indication that the PGEs can be used in the electrochemical analysis of diverse samples.

### 2.9. Data Analysis

Results are presented as the mean ± standard deviation. Data were analyzed using AfterMath^TM^ software and Microsoft^®^ Excel 2013. Statistical analysis across electrode type (e.g., PGE *vs.* GCE,) was performed using two-tailed unequal variance Student’s t-tests, with results deemed significant at *p <* 0.05. To account for differences in the electrode surface area of the GCE and MPE relative to the PGE, all signal area data were divided by the surface area of the respective electrode prior to comparative analysis (surface area = 0.071 cm^2^ for the 0.3 cm diameter GCE; 0.202 cm^2^ for the 0.07 cm diameter, 0.9 cm long MPE; and 0.031 cm^2^ for the 0.2 cm diameter PGEs). Electrode surface areas were calculated as π*r*^2^ (for PGE and GCE) or π*r*^2^ + 2π*r*h (for MPE), where r is the radius and h is the length of the exposed MPE). For instance, if the raw signal area for the MPE in a 8.0 ×10^−3^ M gallic acid standard was 159 µW, then the adjusted area would be 788 µW/cm^2^ (*i.e*., 159 µW ÷ 0.202 cm^2^). All curves were also adjusted to baseline zero.

The limits of detection (LOD) and quantitation (LOQ) of each electrode type were calculated from the GA calibration curves:

LOD = 3 s/m

LOQ = 10 s/m

where *s* is the standard deviation of the intercept and *m* is the slope [32].

## 3. Results and Discussion

### 3.1. Characterization of Electrode Sensors in 1 mM Ferricyanide

**Figure 1:**
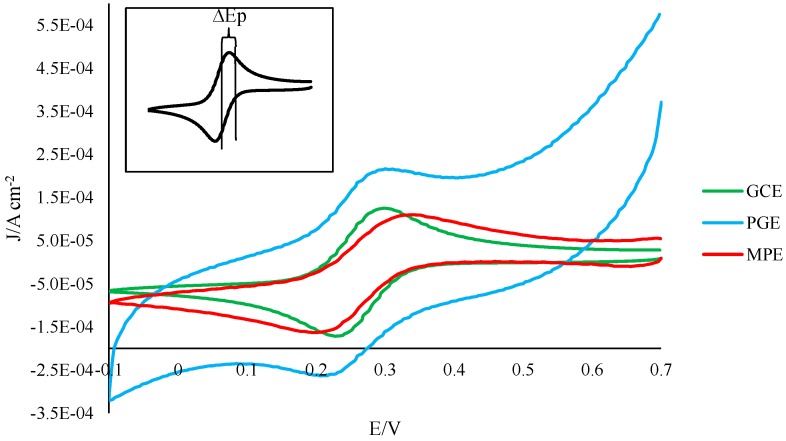
Representative voltammograms of GCE, MPE and PGE electrodes in 1 mM ferricyanide solution. To account for differences in exposed area, current is given as a function of raw current divided by electrode surface area. Initial potential = 0.7 V; vertex potential = −0.1 V; final potential = 0.7 V; scan rate = 0.05 V/s.

A widely accepted method for the electrochemical characterization of surface reactivity is the analysis of electrodes in 1 mM ferricyanide by cyclic voltammetry [16]. The oxidation and reduction peaks of the resultant curve are used to determine the ∆Ep of the electrode (oxidation potential minus reduction potential) (Figure 1). The smaller the ∆Ep, the faster the rate of electron transfer, and the more reactive the surface. Ideally, the ∆Ep for a carbon-based electrode surface should be near 70 mV [16].

To begin the characterization of the developed PGE sensor, all electrode types (GCE, MPE, PGE) were analyzed in 1 mM ferricyanide, and the respective ∆Ep values determined and compared (Table 1). No significant differences were observed between the GCE and PGE, suggesting that the PGE sensor demonstrates electron transfer rates comparable to glassy carbon. Interestingly, however, a significant difference was observed between the GCE and the MPE (*p* = 0.0003), with the GCE possessing a significantly smaller ∆Ep. A significant difference was also observed between the MPE and the PGE; the PGE possessed a significantly smaller ∆Ep. This indicates that, while comparable to the GCE, the developed PGEs are superior to the MPE in terms of electron transfer kinetics. Consequently, the use of PGEs in place of MPEs in electrochemical studies has the potential not only to decrease variability and error (since the same pencil could be used for an entire series of experiments), but could also lead to more accurate signals due to more rapid electron transfer.

**Table 1:** Characterization of electrodes in 1 mM ferricyanide. Initial potential = 0.7 V; vertex potential = −0.1 V; final potential = 0.7 V; scan rate = 0.05 V/s.

Electrode Type	∆Ep (mV) (mean ± standard deviation, n = 3)	P Value (*vs.* GCE)	P Value (*vs.* MPE)
*GCE*	68.9 ± 4.9	-	-
*MPE*	133.8 ± 6.6	0.0003*	-
*PGE*	74.5 ± 3.1	0.1806	0.0010*

* Denotes significant value (*p* < 0.05); - Denotes either not applicable or already stated in an earlier column.

### 3.2. Characterization of Electrode Sensors in Gallic Acid

**Figure 2:**
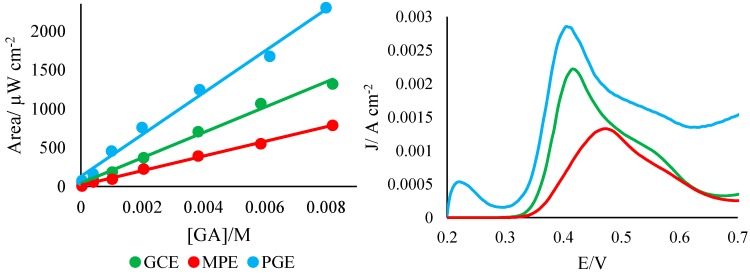
Characterization of PGEs in gallic acid standards. **Left**: calibration curves for electrodes in gallic acid ([GA] = 8 × 10^−3^–4 × 10^−5^ M, n = 4); **Right**: representative voltammograms of electrodes in gallic acid (2 × 10^−3^ M). To account for differences in exposed area, current is given as a function of raw current divided by electrode surface area.

In addition to characterization of electron transfer kinetics in ferricyanide, the reactivity of the developed PGE was assessed using gallic acid (GA) standards (Figure 2). In all cases, the PGE demonstrated responses comparable or superior to those from the GCE. As can be seen in Table 2, the LOD and LOQ of the PGE were on the same order as those of the GCE, and were very near those of the MPE. All electrode types analyzed also demonstrated similar linearities (*r*^2^ ≈ 0.99). However, the PGE demonstrated a much greater sensitivity than either the GCE or MPE, as indicated by the steepness of the PGE curves: the slope of the PGE curve was approximately 1.5 times greater than that of the GCE, and 2.7 times greater than that of the MPE. Furthermore, PGE results were generally more reproducible than those from the GCE or MPE, as indicated by the percent relative standard deviations (% R.S.D.) of the peak potentials and areas. Overall, this suggests that PGE sensors can act as viable, highly sensitive replacements for both GCEs and MPEs in the electrochemical study of antioxidants in natural samples.

**Table 2:** Validation parameters for PGEs. Parameters derived from calibration curve of gallic acid in 0.1 M phosphate buffer at pH 2.0, linear range 8 × 10^−3^ –4 × 10^−5^ M.

Validation Parameter	GCE	MP	PGE
Peak potential (mV)	399.7 ± 10.1	512.0 ± 36.6	404.1 ± 0.32
Correlation coefficient	0.9927	0.9973	0.9900
Slope (µW·cm^2^/mol*)*	163,107 ± 6252	94,389 ± 2204	268,853 ± 12,058
% R.S.D. of slope	3.83	2.34	4.48
Intercept (µW)	41.5 ± 26.1	13.7 ± 9.2	129.7 ± 50.4
Number of solutions	7	7	7
LOD (M)	4.80 × 10^−4^	2.93 × 10^−4^	5.62 × 10^−4^
LOQ (M)	1.60 × 10^−3^	9.75 × 10^−4^	1.87 × 10^−3^
% R.S.D. of potential	2.51	7.15	0.08
% R.S.D of peak area	0.95	13.47	3.38

### 3.3. Analysis of Electrodes in Fruit/Vegetable Extracts

**Figure 3:**
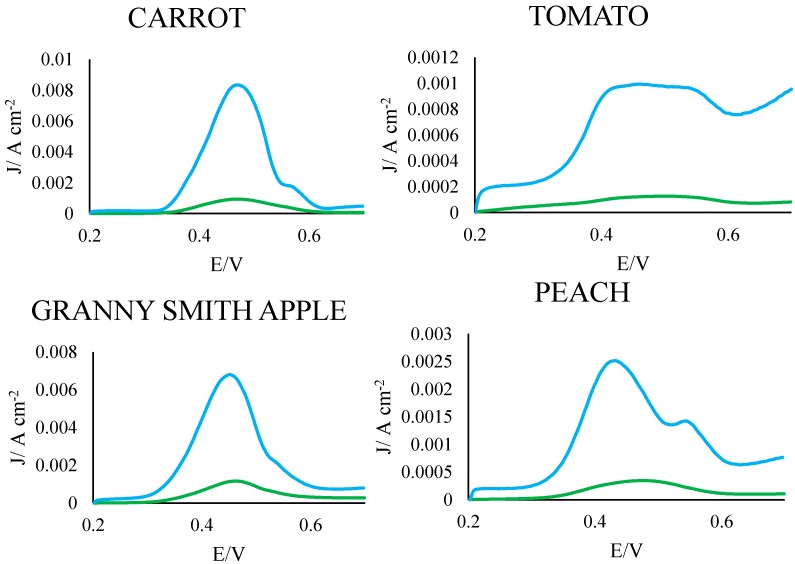
Representative voltammograms of the PGEs (blue) and GCEs (green) tested in aqueous fruit and vegetable extracts. To account for differences in exposed area, current is given as a function of raw current divided by electrode surface area.

To assess their utility as antioxidant sensors in complex biological matrices, PGEs were also analyzed in aqueous extracts of carrot, tomato, apple and peach. For all samples analyzed, the PGE demonstrated a significantly more intense peak and larger signal area than the commercial GCE (Table 3, Figure 3): signal areas and peak heights for the PGE were 4 to 10 times larger than those of the GCE. This increased sensitivity can likely be attributed to the clay (aluminum, silicon and oxygen) constituent of the pencil lead, which has been shown to increase conductivity relative to pure carbon, leading to a more intense signal [16]. This demonstrates that PGEs can not only serve as viable replacements for commercial GCEs in the analysis of standards, but also in the analysis of complex natural matrices.

**Table 3:** Analysis of electrodes in fruit and vegetable extracts.

		Peak Potential (mV)	Peak Height (µA)	Signal Area Per Unit Surface Area (µW/cm^2^)	P Value (Peak Height)	P Value (Signal Area)
Carrot	GCE	470.9 ± 3.0	870.9 ± 166.7	143.2 ± 22.8	0.008 *	0.003 *
	PGE	467.4 ± 1.2	8721.2 ± 1253.0	1227.1 ± 121.0		
Tomato	GCE	498.8 ± 8.2	125.4 ± 4.4	40.3 ± 2.3	2 × 10^−9^ *	9 × 10^−6^ *
	PGE	451.1 ± 13.5	992.6 ± 4.5	342.2 ± 5.4		
Apple	GCE	459.7 ± 3.3	1261.8 ± 85.0	222.3 ± 10.4	0.002 *	0.002 *
	PGE	451.09 ± 0.04	6334.4 ± 487.7	998.9 ± 60.3		
Peach	GCE	476.4 ± 2.6	280.7 ± 66.2	61.0 ± 13.0	0.005 *	0.003 *
	PGE	433.7 ± 0.5	2816 ± 360.3	558.5 ± 55.1		

* Denotes significant value (*p* < 0.05)

### 3.4. Proof of Principle

**Figure 4:**
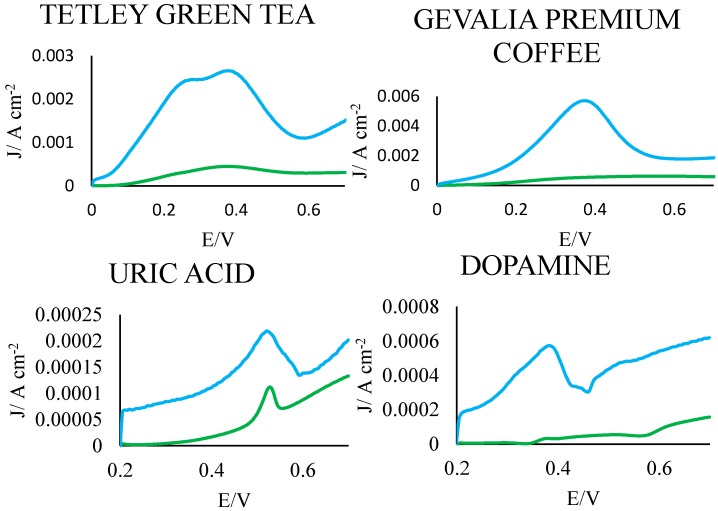
Proof of principle. Representative voltammograms of the PGEs (blue) and GCEs (green) in samples of coffee, tea, UA (0.00001 M) and DA (0.00001 M). To account for differences in exposed area, current is given as a function of raw current divided by electrode surface area.

As further proof of principle, the developed PGEs were used to analyze samples of coffee and tea, as well as samples of the critical biological compounds uric acid (UA), a metabolic end product, and dopamine (DA), a vital neurotransmitter [33]. As with fruit and vegetable extracts, the PGEs consistently demonstrated a dramatically more intense peak and a larger signal area than the GCE in the same solution (Figure 4), further validating the PGE as a cheap, disposable, yet sensitive biological sensor.

### 3.5. Overall Evaluation of the PGE 

In addition to possessing increased sensitivity, the developed PGE sensors demonstrated several structural and logistical advantages over both GCEs and MPEs (Table 4).

**Table 4:** Qualitative comparison of GCEs, MPEs and PGEs.

	Approximate Cost Per Electrode (CDN)	Total Scans Possible with Each Electrode	Total Polishing/Preparation Time Per Scan	Single Electrode Can Be Used for Entire Experimental Series?
GCE	$190	indefinite	15–60 min	Yes
MPE	$0.13	3	10–20 s	No
PGE	$0.30	40–50	30 s–1.5 min	Yes

#### 3.5.1. PGE *vs.* GCE

Though exact preparative methods for GCEs vary slightly, in most cases GCEs must be polished prior to each scan, using a procedure which typically requires three successive treatments with alumina polish and at least four separate sonications. The total time required for this rigorous process can vary from 15 to 60 min, depending upon the particular sequence used. This time requirement quickly accumulates when attempting to run multiple scans in each test solution, such that polishing becomes a key constraint on the maximum rate at which analysis can be carried out. In contrast, the total preparative time for PGEs between scans varies from approximately 30 s (when polishing on sandpaper between analyses in the same solutions) to 1.5 min (when removing the tip between analyses in different solutions), and each PGE can be used for approximately 40–50 scans (compared to each MPE, which could only be used for 2–3 scans). Furthermore, the PGEs are disposable, and as such can be used in all manner of samples.

PGEs were checked regularly in ferricyanide for any alterations in signal occurring as a result of the decrease in length over the course of testing; however, no significant peak shift was observed, indicating that the PGEs can be used along their entire length. Furthermore, because the surface layer of graphite is removed following each scan, no interference from passivation occurs.

We would also like to emphasize that the initial time investment described in Section 2.2 is not per electrode. Rather, several dozen PGEs can be prepared at once: in the space of a single day (the time required for full preparation of the electrodes, including curing of the SG), enough electrodes to last for more than 6 months of full-time research can be prepared. Furthermore, because of the simple polishing requirements of PGEs relative to GCEs, the use of PGEs for analytical analysis can save more than 15 min per scan, leading to more rapid analysis. Thus, despite the initial time investment required for their preparation, in the long term, the use of PGEs leads to a significant time savings.

The developed PGE also demonstrated a very significant reduction in cost relative to the commercial GCEs. SG ($10 per canister) was able to coat approximately 50 PGEs for a cost of $0.30 each. This represents a dramatic savings over the $190+ generally needed to purchase a commercial GCE. Even assuming a total average of only 25 scans per pencil (to account for defective pencils and any breakage which might occur), approximately 600 PGEs could be prepared for the cost of a single GCE, equating to over 15,000 scans.

#### 3.5.2. PGEs *vs.* MPEs

As explained in the introduction, MPEs have been successfully used in numerous electrochemical fields to analyze everything from vitamins [18,19] to drugs [22] to biological samples [23,24,25]. Like the developed PGE, MPEs are simple and rapid to use, and possess many of the prime advantages of pencil graphite, including: disposability, low-cost, electrochemical reactivity, ready availability, and rapid turnover between scans [34]. However, despite their widespread usage, MPEs also possess several drawbacks: they are extremely fragile; due to their short length and fragility, the same lead cannot be used for an entire experimental series, which can introduce error; and it is difficult to ensure that the surface of the lead which is exposed to the solution has not been contaminated through air exposure, or through contact with other surfaces. These disadvantages can be overcome through use of the developed PGE, without loss of the positive aspects of graphite electrodes.

**Figure 5:**
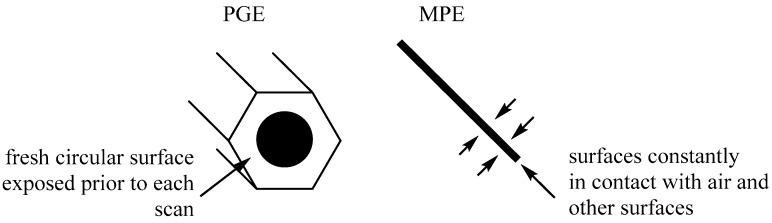
Diagram of PGE *vs.* MPE, highlighting the difference in exposed surface area.

Because the lead of the developed PGE is encased in a hydrophobic wooden tube, the electrodes are structurally stable, and can endure repeated polishing and small impacts without damage, thereby overcoming the fragility limit of the MPEs. Similarly, because the PGEs are more stable and are longer than their mechanical lead counterparts, a single PGE can be used for an entire experimental series, reducing error caused by the use of different electrodes. Finally, because a fresh PGE surface is exposed for every scan, similar to a GCE, contamination through air exposure or contact with surfaces is controlled (Figure 5).

In addition, as revealed in Section 3.1 and Section 3.2, the developed PGE tends to be more sensitive than MPEs. Furthermore, the PGEs tend to demonstrate peak potentials more similar to the GCE (Figure 1 and Figure 2), possibly due to the similarity of the wooden HB#2 PGE’s electron transfer rates to those of the GCE, as described in [16]. Thus, overall, our results represent the developed PGEs as a novel, extremely cost-effective, highly sensitive alternative to traditional GCEs and MPEs.

## 4. Conclusions

In this study, a novel pencil-graphite electrode (PGE) sensor was developed from an HB#2 pencil, and the reactivity of the PGE compared against that of a commercial glassy-carbon electrode (GCE) and a mechanical pencil electrode (MPE). In both standard antioxidant analysis and in the analysis of several biological samples, the PGE sensor consistently demonstrated sensitivities and reproducibilities comparable or superior to those of the GCE and MPE. The PGE was thus shown to be a viable, disposable, and extremely low-cost alternative to traditional carbon electrodes. The developed PGE could thus potentially replace the use of GCEs in electrochemical analyses. Furthermore, if combined with a low-cost potentiostat (~$50) and a low-cost reference electrode (~$1), similar to those developed by Mott *et al*. [35] and Masse and Gerken [36], respectively, these PGEs could enable electrochemical experiments to be carried out at low cost, which is greatly beneficial to all researchers working in the electrochemical sensors field due to the reduced cost of the working electrode.
